# Moving pathogen genomics out of the lab and into the clinic: what will it take?

**DOI:** 10.1186/s13073-015-0254-z

**Published:** 2015-12-30

**Authors:** Leila M. Luheshi, Sobia Raza, Sharon J. Peacock

**Affiliations:** PHG Foundation, 2 Worts Causeway, Cambridge, CB1 8RN UK; London School of Hygiene and Tropical Medicine, London, WC1E 7HT UK

## Abstract

Pathogen genomic analysis is a potentially transformative new approach to the clinical and public-health management of infectious diseases. Health systems investing in this technology will need to build infrastructure and develop policies that ensure genomic information can be generated, shared and acted upon in a timely manner.

## The promise of pathogen genomics

Whole-genome sequencing (WGS) of specific pathogens and metagenomic sequencing of communities of microbes have now been used in a wide range of clinical and epidemiological research investigations of infectious disease. WGS of a pathogen has enabled the resolution of a methicillin-resistant *Staphylococcus aureus* outbreak on a neonatal intensive care ward [1] and the successful identification of sources of *Pseudomonas aeruginosa* infections on a burns unit [2] and, most recently, it has been deployed for the real-time surveillance of the Ebola outbreak in West Africa [3]. Metagenomics has been successfully used to characterise the strain of Shiga-toxin-producing *Escherichia coli* (STEC) O104:H4 that was responsible for a pan-European outbreak [4] and in a diagnostic context to identify rare neurological infections [5] and transplant-related infections [6]. These striking examples of the power of genomics in this field of medicine have sparked considerable excitement within the healthcare sector.

Moves are now afoot within a number of health systems to establish pathogen WGS infrastructure and services to meet the ongoing need to manage infectious disease at a national and international level. In the USA, the Food and Drug Administration has developed GenomeTrakr (http://www.fda.gov/food/foodscienceresearch/wholegenomesequencingprogramwgs/ucm363134.htm), a geographically distributed, multi-agency WGS-based surveillance network for detecting and investigating foodborne disease outbreaks. Similarly, Public Health England is now routinely using WGS at its national microbiology reference laboratory for *Salmonella* infection surveillance and outbreak investigation [6]. Both of these programmes have had notable successes in enhancing the effectiveness of investigations of foodborne infectious disease outbreaks (http://www.cdc.gov/mmwr/preview/mmwrhtml/mm6406a3.htm?s_cid=mm6406a3_e) [7].

The ability of WGS to improve the sensitivity and specificity of such challenging outbreak investigations is acting as a strong driver for the implementation of WGS services by public-health authorities worldwide. Notably, the Global Microbial Identifier project (http://www.globalmicrobialidentifier.org/People), which was established to support the development of an international system of genomics-based infectious disease surveillance, has members from 42 nations.

A major challenge in the field is to find the optimal solutions to bring the benefits of this new technology to patients as quickly, effectively and equitably as possible. These solutions are likely to vary widely between health systems. Where genomics services need to be delivered to large geographically dispersed populations with devolved political and health infrastructures, such as in the USA, a distributed network approach might be the best solution. By contrast, when dealing with smaller populations in countries (such as England) where a strong, centralised nationwide disease surveillance mechanism is in place, building on this existing framework might be more effective. Despite these geographical variations in health-system organisation, some common principles exist, which we have identified through our analysis of pathogen genomics within the context of the English health system [8]. These common principles will need to be considered regardless of location.

## One size does not fit all

It is important that pathogen genomics services are configured in a way that allows for flexibility. Providing genomics infrastructure and skills that are responsive to the diverse needs of clinicians and public-health practitioners is essential, yet not straightforward. Clinical diagnostic or time-sensitive investigations may require low-throughput, ‘on demand’ sequencing and automated analysis — on benchtop or even portable sequencers — to deliver actionable results to clinicians. Portable sequencing kits have, for example, enabled ‘lab in a suitcase’ solutions to be deployed for real-time genomic surveillance of the Ebola virus in West Africa [3]. By contrast, larger scale, longitudinal public health surveillance activities could be delivered using approaches with higher throughput but longer turnaround times. An enormous variation also exists in access to genomic technology and expertise across health systems, as well as variation in the level of the underlying need for genomics as a tool to manage infectious disease. Those charged with management and commissioning of infectious disease services within individual health systems will, therefore, have to remain extremely flexible if they are to navigate this complex landscape of supply, demand and capability. It will be important to avoid over-investing in technology that could rapidly become obsolete and recognise that the optimal configuration and location of genomics services will vary according to local demand and the availability of genomics skills and infrastructure, which are also subject to rapid change.

## Pathogen genomics is a team game

The most successful demonstrations of the capabilities of pathogen genomics were the deciphering of the origins of the Middle East respiratory syndrome coronavirus and the management of the Europe-wide 2011 STEC outbreak. These have also been excellent examples of the effectiveness of collaborations, which were supported by openness and data sharing, that span multiple geographies, fields of expertise and professional groups. As we move towards implementing these advances across entire health systems, this collaborative spirit needs to be harnessed and scaled up to deliver more formalised networks for sharing knowledge and best practice. These networks will expedite national service deployment, accelerate future service development and provide expert forums in which the arduous but essential task of establishing the standards and benchmarks of service quality can be undertaken. High-level strategic coordination and knowledge-sharing across health delivery organisations are equally essential. For example, synergy in the development of pathogen genomics programmes across animal and human health sectors will be needed to deliver a ‘one-health’ approach to tackling many of the most pressing zoonotic disease threats, such as avian influenza and the spread of antimicrobial resistance from livestock to humans, as well as the more commonplace but persistent issue of foodborne illnesses.

## Data — it’s time to go big

No large-scale genomics enterprise can hope to succeed without effective data integration and sharing. In the case of pathogen genomics for clinical or public health, data integration will require the construction of dedicated infrastructure (real or virtual) to collate, store, analyse and share genomic, epidemiological and clinical data across complex national and international health systems. This integration will be particularly crucial to enable the delivery of genomic epidemiology services, in which the requirement for data sharing across locations is fundamental and time (as in the 2015 Ebola outbreak) is very much of the essence. Data integration and access will become equally central in diagnostic microbiology, where having an accurate and readily accessible summary of genotype, phenotype and clinical information for different pathogens is essential to deliver care to patients.

Notably, the Global Microbial Identifier (http://www.globalmicrobialidentifier.org/Workgroups#work-group-1) and the Global Alliance for Genomic Health (https://genomicsandhealth.org/node/12703) projects are already demonstrating that huge technical, regulatory and political efforts will be required to overcome barriers imposed by the varying capabilities, legal systems and cultural frameworks of different nations if transnational genomic and clinical data integration is to be achieved. As an example, such barriers led the Indonesian government to temporarily withdraw from the Global Influenza Surveillance Network in 2007, owing to concerns that, if data were shared, proprietary interests would be exerted over the H5N1 strains [9]. The same issues can also impose barriers to sharing and data integration within individual health systems.

## Staying ahead of the game

The application of genomics to infectious disease management is still in its infancy, and we currently lack the background knowledge, as well as the technological capability, to deliver rapid, clinically actionable information based on genomic analysis that can improve outcomes for patients. This situation is fluid, as sequencing technology (in the form of devices that are increasingly more portable, cost less and have a longer read capability) and analysis (in the form of cloud computing, more efficient metagenomic analysis and implementation of automated analytic pipelines) continue to develop apace. However, capitalising on the current rapid pace of innovation will demand novel approaches to public–private cooperation and co-development of the type seen with the Oxford Nanopore Minion Access Programme, which puts new genomic technology in the hands of its users (including those in the health system) at the earliest possible opportunity [10]. These efforts will also benefit from building on the commendably open and collaborative approaches for the development of knowledge and analytical methods that are already being taken by many pathogen genomics researchers. These collaborations will ensure that the knowledge and expertise of these researchers are shared rapidly with the clinical and public-health communities in which their influence is most needed.

Most importantly, health systems must adapt to the reality of rapid innovation in genomics. Current approaches to implementation of innovation in health systems are often characterised by pilots, trials and regulatory hurdles so numerous that by the time they are completed the technology is already becoming obsolete. These approaches must be replaced with more agile and streamlined approaches that are properly resourced and that empower clinicians and scientists to operate services that are being continuously updated and improved in light of new knowledge and technology.

## Conclusions

The path to successful pathogen genomic service delivery in the health system is likely to be interrupted by a number of potentially unpredictable step-changes in technological innovation and discovery. It is vital that researchers remain engaged in the process of frontline implementation over the coming years. Effective collaborations with healthcare practitioners and policy-makers will be central to catalysing the transformation of these innovations from inspirational case studies to the core of a widely available and routinely delivered genomics-enabled system of infectious disease management.

## Abbreviations

STEC: Shiga toxin-producing *Escherichia coli*
WGS: Whole-genome sequencing
